# Efficacy of Dextromethorphan Augmentation in SSRI‐Resistant Obsessive–Compulsive Disorder: A Randomized Controlled Trial

**DOI:** 10.1002/hsr2.72055

**Published:** 2026-05-05

**Authors:** Ahmad Zolghadriha, Afagh Anjomshoaa, Romina Bagheri

**Affiliations:** ^1^ Department of Psychiatry, Shahid Beheshti Hospital Zanjan University of Medical Sciences Zanjan Iran; ^2^ Zanjan Metabolic Diseases Research Center Zanjan University of Medical Sciences Zanjan Iran

**Keywords:** dextromethorphan, obsessive–compulsive disorder, randomized clinical trial, SSRIs

## Abstract

**Background and Aims:**

Obsessive–compulsive disorder (OCD) is a chronic condition associated with marked functional impairment and high rates of treatment resistance. Converging evidence implicates glutamatergic dysregulation in OCD pathophysiology. Dextromethorphan (DXM), an N‐methyl‐d‐aspartate (NMDA) receptor antagonist with additional monoaminergic and sigma‐1 receptor activity, may modulate this system. This study evaluated the safety and efficacy of DXM augmentation in adults with moderate to severe OCD receiving stable SSRI treatment.

**Methods:**

In this randomized, double‐blind, placebo‐controlled pilot trial 40 adults with moderate to severe OCD (Y‐BOCS > 16) and inadequate response to at least 12 weeks of adequate‐dose SSRI monotherapy were assigned to DXM (15 mg twice daily) or matched placebo, in addition to their ongoing SSRI, for 12 weeks. OCD symptom severity was assessed using the Yale–Brown Obsessive Compulsive Scale (Y‐BOCS) at baseline, week 4, and week 12. Repeated‐measures analysis of variance (ANOVA) was used to examine changes over time. The primary outcome was the time × group interaction in Y‐BOCS. Secondary outcomes were not assessed. Trial registration: IRCT20221227056943N1; Ethics approval: IR.ZUMS.REC.1401.236.

**Results:**

Baseline demographic and clinical characteristics were similar between groups. The DXM group showed a significant reduction in Y‐BOCS scores from 26.55 ± 6.19 at baseline to 16.30 ± 6.94 at week 12, whereas the placebo group remained relatively unchanged. Repeated‐measures ANOVA revealed a significant group × time interaction (*p* < 0.001, partial *η*² = 0.662) and significant main effects of both group (*p* = 0.042) and time (*p* < 0.001). DXM was well tolerated with no side effects. No secondary outcomes were measured in this pilot study.

**Conclusion:**

Dextromethorphan augmentation significantly reduced OCD symptom severity in patients with partial response to SSRIs, supporting its potential role as an adjunctive treatment. These findings warrant larger, longer‐term trials to confirm efficacy, determine optimal dosing, and clarify neurobiological mechanisms.

## 1 Introduction

Obsessive–Compulsive Disorder (OCD) is a chronic and debilitating neuropsychiatric disorder characterized by the presence of obsessions (intrusive, unwanted thoughts) and/or compulsions (repetitive behaviors aimed at reducing distress) [1]. OCD significantly impairs multiple aspects of an individual's life, including occupational functioning, academic performance, interpersonal relationships, and overall quality of life [2, 3, 4]. Beyond its psychological burden, OCD exerts a considerable socioeconomic impact, including reduced productivity, increased healthcare utilization, and strain on family systems [5, 6]. The World Health Organization (WHO) has ranked OCD among the top 10 most disabling medical conditions worldwide in terms of lost income and diminished quality of life [7]. The lifetime prevalence of OCD is estimated to be between 2% and 3% globally, with similar rates reported across different cultures and populations [8].

Neuropsychological and cognitive studies have revealed a range of affective processing abnormalities, and executive function deficits in OCD, including cognitive inflexibility, motor impulsivity, excessive habitual responding, disgust sensitivity, and abnormal reward‐related decision‐making [9, 10]. Structural and functional imaging studies have consistently implicated the cortico‐striatal‐thalamo‐cortical circuitry (CSTC), particularly involving the anterior cingulate cortex, orbitofrontal cortex, and striatum [11, 12]. Alterations in gray matter volume and white matter connectivity within these circuits suggest a neurodevelopmental basis for the disorder [13].

Clomipramine was the first approved treatment for OCD, but due to its side effects, it was largely replaced by selective serotonin reuptake inhibitors (SSRIs), which offer similar efficacy with better tolerability [14]. Nevertheless, 40%–60% of patients show only partial or minimal response to these standard treatments. Cognitive‐behavioral therapy (CBT), particularly exposure and response prevention, remains the most effective non‐pharmacological intervention. Additional psychosocial approaches, including intensive residential treatment programs, family‐based interventions, and metacognitive therapy, may provide benefit in selected cases [15, 16]. However, many patients require adjunctive strategies to achieve meaningful remission. Psychodynamic therapies lack strong empirical support, while advanced interventions like deep brain stimulation (DBS), repetitive transcranial magnetic stimulation (rTMS), and ablative neurosurgery are reserved for severe, treatment‐resistant cases [17, 18, 19].

Augmentation of SSRIs with atypical antipsychotics, particularly risperidone and aripiprazole, is currently the best‐established pharmacological strategy for treatment‐resistant OCD, with multiple randomized controlled trials demonstrating modest to moderate efficacy in a subset of patients. However, antipsychotic augmentation is limited by significant metabolic adverse effects, extrapyramidal symptoms, hyperprolactinemia, and concerns regarding long‐term safety. Furthermore, a substantial proportion of patients fail to respond to or cannot tolerate antipsychotic augmentation, underscoring the need for alternative adjunctive agents with different mechanisms of action and more favorable tolerability profiles.

Recent research has identified glutamatergic dysregulation as a key component in the pathophysiology of OCD [20]. Glutamate is the principal excitatory neurotransmitter in the central nervous system and a precursor to both GABA and glutathione [20]. Studies involving cerebrospinal fluid (CSF) analysis have demonstrated elevated glutamate concentrations in un‐medicated OCD patients, while magnetic resonance spectroscopy (MRS) studies have shown increased glutamate in the basal ganglia and reduced levels in the anterior cingulate cortex [21, 22]. Also, genetic studies further support glutamatergic involvement through polymorphisms in genes regulating glutamate metabolism and signaling. Consequently, glutamate‐modulating drugs like memantine, N‐acetylcysteine (NAC), riluzole, D‐cycloserine, and ketamine have been studied, though most have yet to become common clinical treatments [20, 23].

Dextromethorphan (DXM), a commonly available cough suppressant, is now under investigation due to its N‐methyl‐D‐aspartate (NMDA) receptor antagonist properties, as well as its modulation of serotonin, norepinephrine, nicotinic acetylcholine, and sigma‐1 receptors [24, 25]. When used in combination with metabolic inhibitors like bupropion or quinidine, its central nervous system effects are potentiated [26]. DXM has shown efficacy in treating major depressive disorder (MDD), another condition involving CSTC dysfunction, and is generally better tolerated than antipsychotic augmentations used in OCD [24]. The overlapping neurobiological mechanisms between MDD and OCD, including CSTC circuit abnormalities and glutamatergic dysregulation, support the rationale for investigating DXM as an adjunctive treatment in OCD [27].

The current study is designed to assess the safety and effectiveness of DXM augmentation in patients with moderate to severe OCD already receiving stable SSRI doses. Using a randomized, double‐blind, placebo‐controlled design, the trial aims to determine whether DXM can produce greater symptom reduction and functional improvement compared to placebo, offering a promising new option in the treatment of refractory OCD.

## 2 Materials and Methods

### 2.1 Study Design and Setting

This randomized, double‐blind, placebo‐controlled pilot trial was conducted to evaluate the effect of dextromethorphan on OCD symptoms in patients with moderate to severe OCD. The study was carried out at Shahid Beheshti Hospital, Zanjan, Iran, from January 2023 to March 2024. The research protocol was approved by the Ethics Committee of Zanjan University of Medical Sciences (Ethics approval: IR.ZUMS.REC.1401.236) and was registered in the Iranian Registry of Clinical Trials (Trial registration: IRCT20221227056943N1), and full registration details are available on the IRCT website (https://www.irct.ir). The study adhered to the Consolidated Standards of Reporting Trials (CONSORT) guidelines for randomized controlled trials. Written informed consent was obtained from all participants after explaining the study objectives, procedures, benefits, and risks. Participation was voluntary, and confidentiality of participants' information was strictly maintained throughout the study.

### 2.2 Inclusion and Exclusion Criteria

The inclusion criteria for the study are as follows: age over 18 years, a diagnosis of obsessive–compulsive disorder (OCD) according to DSM‐5 criteria with moderate to severe severity (Y‐BOCS score > 16), inadequate response to at least 12 weeks of treatment with a standard‐dose SSRI (fluoxetine 40–80 mg/day, sertraline 150–200 mg/day, paroxetine 40–60 mg/day, fluvoxamine 200–300 mg/day, or escitalopram 20–30 mg/day) at doses within the therapeutic range typically used for OCD, and signed informed consent. Inadequate response to SSRI treatment was defined as persistence of at least moderate OCD symptom severity (Y‐BOCS total score > 16) after a minimum of 12 weeks of adequate‐dose SSRI treatment, together with less than 35% reduction in Y‐BOCS score from the pre‐SSRI baseline (where available) or ongoing clinically significant functional impairment despite treatment. Patients with comorbid psychiatric disorders (except for major depressive disorder), severe renal or hepatic impairment, pregnancy or breastfeeding, unstable medical conditions, and a history of allergy or intolerance to dextromethorphan were not included in this study. Exclusion criteria included allergy or hypersensitivity to DXM, pregnancy, and unwillingness to continue participating in the study. At baseline, we also recorded the number of prior adequate SSRI trials and whether participants had previously received cognitive‐behavioral therapy or exposure and response prevention. Furthermore, none of the participants had previously completed a full course of cognitive‐behavioral therapy (CBT) or exposure and response prevention (ERP), and no participants were receiving CBT during the study period. Details of SSRI agents and dose ranges in each group are provided in Supporting Information Table S1.

### 2.3 Participant Selection and Sampling

As a pilot study with a small sample size, this trial aimed to generate preliminary effect size estimates to guide the design of future, adequately powered randomized trials. Outpatients and inpatients diagnosed with OCD who met the inclusion criteria were recruited consecutively from Shahid Beheshti Hospital. Initially, 49 patients were enrolled; however, after dropouts, 40 participants (20 per group) completed the study. Randomization was performed using a table of random numbers, with even numbers assigned to the intervention group and odd numbers to the control group, continuing until the target sample size was achieved.

### 2.4 Randomization, Allocation Concealment, and Blinding

Randomization was performed using a computer‐generated random number sequence by an independent researcher who was not involved in the recruitment, treatment, or evaluation phases of the study. Eligible participants were randomly assigned to either the intervention group (dextromethorphan plus SSRI) or the control group (placebo plus SSRI) in a 1:1 ratio. This random allocation ensured an equal probability of assignment to each group and minimized selection bias. To maintain allocation concealment, sequentially numbered, sealed, opaque envelopes were used. Each envelope contained a group assignment and was opened only after a participant had been enrolled and met all inclusion criteria. This method prevented investigators and participants from predicting upcoming allocations, thereby preserving the integrity of the randomization process. Blinding was maintained throughout the study. Both participants and clinical staff, including psychiatrists and outcome assessors, were blinded to group assignments. The placebo tablets were formulated and packaged by the hospital pharmacy to be identical in appearance, shape, color, and packaging to the dextromethorphan tablets. All study medications were labeled with neutral codes to ensure the blinding of both participants and investigators.

### 2.5 Interventions

Participants were randomly assigned to one of two groups, including an intervention group receiving dextromethorphan and a control group receiving placebo. All participants continued their existing treatment with a selective serotonin reuptake inhibitor (SSRI), which had been administered at a stable and clinically adequate dose for at least 12 weeks prior to enrollment. SSRI therapy was maintained unchanged throughout the duration of the study to ensure the effects of the adjunctive intervention could be accurately evaluated.

The intervention group received 15 mg of dextromethorphan orally, every 12 h (twice daily), for a total treatment period of 12 weeks. Dextromethorphan tablets were dispensed by the hospital pharmacy and administered in addition to the participant's ongoing SSRI medication. The control group received placebo tablets identical in appearance, packaging, and administration schedule to the dextromethorphan tablets, also for a period of 12 weeks, alongside their unchanged SSRI regimen. All study medications were coded and prepared by the hospital pharmacy to maintain blinding. Patients, psychiatrists, and outcome assessors remained blinded to treatment allocation throughout the study. The dose of 15 mg twice daily (30 mg/day total) was selected based on several considerations. This dose corresponds to the lower‐to‐middle range of standard antitussive dosing (15–30 mg every 4–6 h as needed) and was chosen to maximize tolerability in this initial proof‐of‐concept trial. Prior psychiatric studies have indicated that DXM at similar or higher daily doses (20–120 mg/day) demonstrates safety and potential efficacy in conditions such as major depressive disorder, pseudobulbar affect, and substance use disorders. The twice‐daily schedule was selected to provide steady‐state coverage while minimizing pill burden.

The 12‐week trial duration was chosen to allow sufficient time to observe the emergence, stability, and potential maintenance of treatment response, consistent with the time course typically required for pharmacological interventions in OCD, and based on the existing literature [28, 29]. This duration also aligns with prior augmentation trials in OCD, which have ranged from 8 to 16 weeks. While the placebo group received inactive augmentation for the full 12‐week period, all participants remained on active SSRI treatment throughout, and those showing significant worsening or distress were afforded the option to discontinue and receive alternative treatment, thereby mitigating ethical concerns. The study was approved by the institutional ethics committee with these safeguards in place.

Participants were evaluated at baseline, week 4, and week 12. At each follow‐up visit, adherence to treatment was assessed through patient self‐report and pill count. Participants were systematically monitored for adverse events using structured clinical interviews and were encouraged to report any new symptoms or concerns between visits. No additional psychotropic medications were introduced, and non‐pharmacological interventions such as psychotherapy were withheld during the study period to reduce potential confounding variables.

### 2.6 Outcome Measures

The primary outcome of the present study was the change in obsessive–compulsive symptom severity, measured by the Yale–Brown Obsessive Compulsive Scale (Y‐BOCS), a widely validated, clinician‐administered, semi‐structured instrument used to assess both the presence and intensity of OCD symptoms. Y‐BOCS assessments were conducted at 3 time points: baseline (defined as 24 h prior to the first intervention dose), week 4, and week 12 following the initiation of treatment. All assessments were performed by psychiatrists blinded to group assignments in order to reduce assessment bias. To maintain consistency, the same blinded rater evaluated each participant across all time points. A clinically meaningful response to treatment was defined as a ≥ 35% reduction in total Y‐BOCS score from baseline. In addition to measuring symptom severity, participants were monitored for adverse events, medication adherence, and any emerging psychiatric or medical complications throughout the 12‐week follow‐up period. While global measures such as the Clinical Global Impressions–Severity and Improvement scales (CGI‐S/CGI‐I) are commonly used in OCD trials as secondary outcomes, they were not systematically administered in the current pilot study due to resource constraints and the decision to prioritize a focused, disorder‐specific primary outcome. Future larger trials should incorporate such global outcome measures to provide a more comprehensive assessment of treatment response. Secondary outcomes were not assessed in this pilot study but are planned for evaluation in subsequent larger trials. All participants were monitored for adverse events at each study visit using structured clinical interviews.

### 2.7 Data Collection Tools

#### 2.7.1 Socio‐Demographic Checklist

Demographic and clinical information were collected at baseline using structured forms. The Y‐BOCS scale, validated for the Iranian population, was used to evaluate symptom severity throughout the study.

#### 2.7.2 Yale–Brown Obsessive Compulsive Scale (Y‐BOCS)

The Yale–Brown Obsessive Compulsive Scale (Y‐BOCS), originally developed by Goodman et al., is a clinician‐rated, semi‐structured instrument comprising 10 items (five assessing obsessive thoughts and five evaluating compulsive behaviors). Each item is scored on a 5‐point Likert scale ranging from 0 (no symptoms) to 4 (extreme symptoms), yielding a total score between 0 and 40. The scale evaluates multiple dimensions of OCD symptoms, including severity, frequency, interference with daily functioning, resistance against symptoms, and perceived control. According to established clinical guidelines, total Y‐BOCS scores are interpreted as follows: 0–7 (subclinical), 8–15 (mild), 16–23 (moderate), 24–31 (severe), and 32–40 (extreme OCD). The psychometric properties of the Persian version of Y‐BOCS have been well documented [30].

### 2.8 Statistical Analysis

Descriptive statistics, including frequencies and percentages for categorical variables and means with standard deviations for continuous variables, were calculated for baseline demographic and clinical characteristics. The Kolmogorov–Smirnov test was applied to assess the normality of the continuous variables. Effect sizes were calculated to quantify the magnitude of between‐group differences at baseline. Depending on the distributional assumptions, between‐group comparisons at baseline were performed using independent‐samples *t*‐tests or Mann–Whitney *U* tests for continuous variables and *χ*² or Fisher exact tests for categorical variables. To examine changes in OCD symptom severity over time, a repeated‐measures analysis of variance (ANOVA) was conducted with time (baseline, week 4, and week 12) as the within‐subject factor and treatment group (DXM vs. placebo) as the between‐subject factor; the time × group interaction on Y‐BOCS scores was pre‐specified as the primary outcome. Partial eta squared (*η*²) was reported as an effect size index for the ANOVA effects. Partial eta squared (*η*²) was reported as a measure of effect size, indicating the proportion of variance in Y‐BOCS scores explained by group, time, and their interaction, providing an estimate of the magnitude of the observed effects. There were no missing data for any variable at any assessment point. All statistical tests were two‐tailed, with a significance level of *α* = 0.05 and 95% confidence intervals where appropriate. Data analysis was carried out using SPSS software, version 24 (IBM Corp., Armonk, NY, USA). No formal a priori power calculation was conducted for this pilot trial. The sample size of 40 participants (20 per group) was determined based on feasibility considerations for this initial proof‐of‐concept study. As such, the trial should be interpreted as generating preliminary effect size estimates to inform the design of adequately powered confirmatory trials rather than as providing definitive evidence of efficacy. Assumptions for repeated‐measures ANOVA (including sphericity, tested by Mauchly's test) were examined; when sphericity was violated, Greenhouse–Geisser corrections were applied. Observed (non‐adjusted) means are reported for descriptive purposes, whereas ANOVA results are based on model‐estimated effects. Post hoc pairwise comparisons were conducted using Bonferroni‐adjusted *p*‐values.

## 3 Results

**Figure 1:**
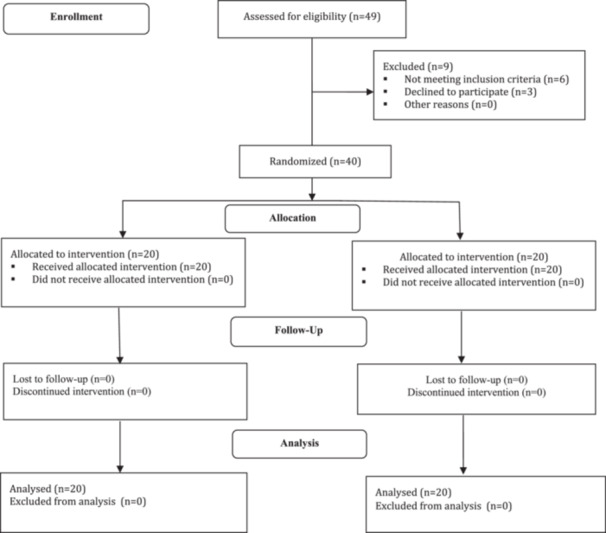
The CONSORT flow diagram of intervention in the two groups.

At the beginning of the study, 49 patients were invited to participate. Of these, six individuals were excluded due to not meeting the inclusion criteria (e.g., comorbid psychiatric or medical conditions, use of disallowed concurrent psychotropic medications (only SSRI monotherapy was permitted)), and three patients declined to participate. Ultimately, 40 eligible participants were enrolled and randomly assigned into two equal groups: intervention and control. The intervention group received dextromethorphan 15 mg twice daily in addition to their stable SSRI regimen, while the control group received a matched placebo. Both groups completed the 12‐week follow‐up, and no missing data or loss to follow‐up occurred. Follow‐up assessments were conducted at baseline, week 4, and week 12. There were no losses to follow‐up in either group, and all participants completed the study. Furthermore, no adverse events or side effects of any severity were reported in either group (0% in both the DXM and placebo arms), indicating that the treatment was well tolerated and safe throughout the trial period (Figure 1).

Demographic characteristics were similar between the intervention and control groups. The mean age of participants in the intervention group was 36.70 years (SD = 10.25), compared to 36.40 years (SD = 13.17) in the control group (*p* = 0.955). The mean duration of illness was 7.70 years (SD = 8.27) in the intervention group and 4.10 years (SD = 2.29) in the control group, which was not statistically significant (*p* = 0.21). No significant differences were found between groups in terms of gender distribution (*p* = 0.11), educational level (*p* = 0.08), or marital status (*p* = 0.33) (Table 1).

**Table 1:** Demographic characteristics of participants in the intervention and control groups.

Variable	Category	Intervention group (*n* = 20)	Control group (*n* = 20)	*p* value
Age (years)	—	36.70 ± 10.25	36.40 ± 13.17	0.955
Duration of illness	—	7.70 ± 8.27	4.10 ± 2.29	0.213
Gender	Male	6 (30%)	12 (60%)	0.110
	Female	14 (70%)	8 (40%)	
Education level	High school	4 (20%)	10 (50%)	0.080
	Diploma	10 (50%)	4 (20%)	
	University	6 (30%)	6 (30%)	
Marital status	Single	6 (30%)	10 (50%)	0.330
	Married	14 (70%)	10 (50%)	

Details of the specific SSRIs (drug name and daily dose range) prescribed to participants in each group at baseline are presented in Supporting Information Table S1.

**Figure 2:**
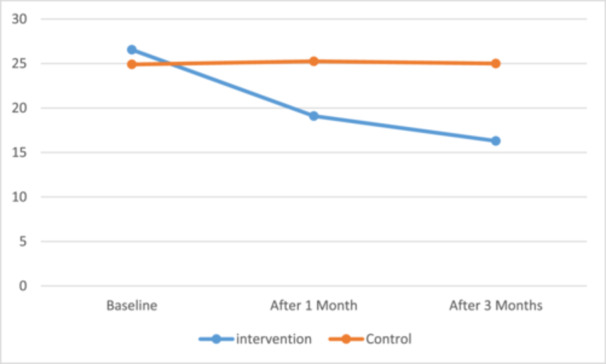
Y‐BOCS scores at baseline, 1 month, and 3 months after the intervention in the intervention and control groups.

At baseline, the mean Y‐BOCS scores were 26.55 (SD = 6.19) in the intervention group and 24.90 (SD = 7.22) in the control group, with no statistically significant difference between the two groups (*p* = 0.565). After 1 month, the mean score in the intervention group decreased markedly to 19.10 (SD = 7.07), while the control group remained relatively unchanged at 25.25 (SD = 6.76). This difference was statistically significant (*p* = 0.035). After 3 months, the intervention group showed a further reduction to 16.30 (SD = 6.94), compared with 25.00 (SD = 6.62) in the control group, representing a significant difference (*p* = 0.001) (Table 2 and Figure 2).

**Table 2:** Mean and standard deviation of Y‐BOCS scores at baseline, 1 month, and 3 months after intervention.

	Intervention	Control	*p* value
Baseline	26.55 (6.19)	24.90 (7.22)	0.565
After 1 month	19.10 (7.07)	25.25 (6.76)	0.035
After 3 months	16.30 (6.94)	25.00 (6.62)	0.001

To examine the effect of DXM augmentation treatment on OCD symptoms, a repeated measure analysis of variance (ANOVA) was conducted. The normality of the distribution of the dependent variable (Y‐BOCS scores) was assessed using the Shapiro–Wilk tests, which confirmed normal distributions. Mauchly's test of sphericity was statistically significant (*W* = 0.626, *χ*² = 17.351, *p* < 0.001), indicating that the assumption of sphericity was violated. Therefore, the Greenhouse–Geisser correction (*ε* = 0.728) was applied to adjust the degrees of freedom for the within‐subjects effects. Additionally, Levene's test showed no significant differences in variances across the 3 time points (*p* > 0.05), confirming the assumption of homogeneity of variances.

The analysis included time (baseline, 1 month, and 3 months) as a within‐subject factor and group (intervention vs. control) as a between‐subject factor. The main effect of group was statistically significant (F (1, df) = 4.424, *p* = 0.042), with a partial eta squared of 0.536, indicating that group membership accounted for approximately 53.6% of the variance in Y‐BOCS scores. There was also a significant main effect of time (F (1.455, df) = 69.327, *p* < 0.001, partial eta squared = 0.646), suggesting a general change in symptom severity over time across both groups. Importantly, the time × group interaction was highly significant (F (1.455, df) = 74.324, *p* < 0.001, partial eta squared = 0.662), indicating that the pattern of change differed between the two groups. Figure 2 shows mean Y‐BOCS scores over time with standard deviation error bars. Post hoc comparisons showed that the intervention group experienced a consistent reduction in Y‐BOCS scores from a baseline mean of 26.55 (SD = 6.19) to 19.10 (SD = 7.07) at 1 month, and further to 16.30 (SD = 6.94) at 3 months, whereas the control group's scores remained relatively stable across time (Table 3).

Due to the small sample size and pilot nature of the study, we did not conduct formal subgroup or multivariable analyses to examine potential moderators of treatment response (e.g., age, sex, baseline symptom severity, specific SSRI type or dose, prior treatment history, or OCD symptom dimensions). Such analyses should be prioritized in future adequately powered trials.

**Table 3:** Results of ANOVA for group differences in OCD symptom severity.

Source of variation	Sum of squares	df	Mean square	F	*p* value	Partial eta squared
Group	580.800	1	580.800	4.424	0.042	0.536
Time	542.450	1.455	372.756	69.327	< 0.001	0.646
Time × group interaction	581.550	1.455	399.624	74.324	< 0.001	0.662

## 4 Discussion

The current study demonstrated a significant difference between dextromethorphan and placebo in reducing the severity of obsessive–compulsive symptoms in patients with moderate to severe OCD who showed an inadequate response to SSRIs. This effect was observed in the primary outcome (Y‐BOCS scores) and remained stable throughout the 12‐week intervention, indicating a sustained therapeutic benefit of DXM. These results are particularly important given the high rate of treatment resistance in OCD, where an estimated 40%–60% of patients fail to achieve remission with first‐line pharmacologic treatments alone [31]. Notably, the therapeutic effects in our study occurred in the absence of concurrent changes in psychotropic medications or psychotherapeutic interventions, strengthening the attribution of observed improvements to the pharmacological properties of DXM.

To our knowledge, this is the first randomized, double‐blind, placebo‐controlled trial to investigate DXM augmentation in adults with OCD, and it provides preliminary empirical evidence for the potential efficacy of targeting glutamatergic mechanisms in SSRI‐resistant illness for the efficacy of glutamate‐modulating strategies in OCD treatment. Previous trials investigating glutamatergic agents, such as riluzole, N‐acetylcysteine (NAC), and pregabalin have shown either modest or inconsistent effects [32, 33]. Pittenger et al. (2015) evaluated riluzole as an adjunct to serotonin reuptake inhibitors (SRIs) but failed to show statistically significant superiority over placebo despite a numerical trend favoring riluzole [28]. Similarly, Costa et al. (2017) examined NAC at a daily dose of 3000 mg in SSRI‐refractory OCD patients and found no significant difference in Y‐BOCS score compared to placebo [29]. Another comparable study by Mowla et al. investigated pregabalin augmentation in OCD and reported greater symptom reduction in the treatment group compared to placebo. However, pregabalin's sedative profile and potential for dependence may limit its broad applicability [29]. By comparison, DXM is widely available, cost‐effective, and generally well tolerated at therapeutic doses, providing it with a practical advantage in clinical settings.

The pharmacological mechanism of DXM likely plays a pivotal role in its observed clinical efficacy in the treatment of obsessive–compulsive disorder (OCD). DXM is a morphinan compound and a non‐competitive antagonist of the N‐methyl‐D‐aspartate (NMDA) receptor, sharing structural and functional similarities with memantine; a compound previously investigated in depression disorder [24]. By antagonizing NMDA receptors, DXM modulates hyperactive glutamatergic neurotransmission within the cortico‐striato‐thalamo‐cortical (CSTC) circuitry, a neuroanatomical substrate extensively implicated in the pathophysiology of OCD [34]. Within this circuit, the striatum, a core component, receives excitatory glutamatergic input from cortical regions, and dysregulation of this pathway has been associated with compulsive behaviors [34, 35]. Beyond NMDA antagonism, DXM exhibits a broader pharmacodynamic profile, including mild inhibition of serotonin and norepinephrine reuptake, as well as agonist activity at sigma‐1 receptors, mechanisms linked to anxiolytic and antidepressant effects [25]. Notably, sigma‐1 receptors function as modulators of neuroplasticity and neuroprotection, potentially enhancing synaptic resilience under stress [34]. Therefore, these multimodal receptor interactions may underlie the therapeutic effects of DXM in attenuating both obsessive and compulsive symptoms, particularly among patients with comorbid mood disturbances.

The study demonstrates several notable methodological strengths, including a randomized, double‐blind, placebo‐controlled design with rigorous blinding procedures that enhance internal validity. Despite these strengths, several limitations must be acknowledged. The small sample size (*n* = 40) restricts the generalizability of the findings and reduces statistical power to detect smaller but clinically meaningful effects. The fixed dosing regimen (15 mg twice daily), selected primarily for tolerability, may not represent the optimal therapeutic dose; future studies should incorporate dose‐ranging designs to clarify dose‐response relationships. The absence of biomarker assessments, such as neuroimaging or serum glutamate measurements, limits mechanistic insights into DXM's effects on CSTC circuitry and glutamatergic modulation. Additionally, the relatively short follow‐up period (12 weeks) limits conclusions regarding long‐term safety, tolerability, and relapse rates. Variability in DXM metabolism via the cytochrome P450 2D6 (CYP2D6) enzyme may also influence central nervous system bioavailability and clinical response. Several additional limitations warrant consideration. First, we did not systematically analyze the potential moderating effects of specific SSRI type or dose, which may be relevant given that potent CYP2D6‐inhibiting SSRIs such as fluoxetine and paroxetine can substantially increase plasma concentrations of DXM by reducing its metabolism to dextrorphan. This pharmacokinetic interaction could result in variable DXM exposure across participants depending on their concomitant SSRI, potentially influencing both efficacy and tolerability. Future trials should stratify by SSRI type or employ pharmacokinetic monitoring. Second, there was a numerical imbalance in sex distribution between groups, with more female participants randomized to the DXM arm. Although baseline Y‐BOCS scores did not differ significantly, sex‐related differences in treatment response, DXM metabolism, or placebo response cannot be ruled out and should be examined in larger, balanced samples. Third, we did not assess OCD symptom dimensions (e.g., contamination/washing, checking, symmetry/ordering, and hoarding) or predominant symptom subtypes, which have been shown in some studies to predict differential treatment response. Fourth, we relied solely on the Y‐BOCS as our outcome measure. The absence of global clinician‐rated measures such as the Clinical Global Impressions–Improvement and Severity scales (CGI‐I/CGI‐S), which are commonly used alongside the Y‐BOCS in OCD trials, limits our ability to assess overall clinical improvement beyond OCD‐specific symptoms. Fifth, the small sample size precluded meaningful subgroup or covariate analyses to identify predictors or moderators of response, such as age, baseline severity, duration of illness, or number of prior treatment failures. Finally, although no serious adverse events were observed and no participants discontinued due to side effects, we did not perform formal blinding‐integrity checks (e.g., asking participants and raters to guess treatment allocation at study end). The absence of differential side effects between groups likely reduced the risk of unblinding, but this cannot be definitively confirmed.

## 5 Conclusion

The results of this study support the use of dextromethorphan as a promising adjunctive treatment in OCD, especially for patients who fail to respond adequately to SSRIs. Through its NMDA antagonism and broader neurochemical effects, DXM may exert therapeutic benefits by modulating hyperactive CSTC circuits and correcting underlying glutamatergic dysregulation. While the findings are encouraging, further research with larger samples, longer follow‐up periods, and incorporation of biological markers is essential to validate these preliminary results. Nonetheless, this study contributes significantly to the emerging evidence base supporting glutamate‐targeted pharmacotherapy in OCD.

## Author Contributions

Romina Bagheri, Afagh Anjomshoaa, and Ahmad Zolghadriha contributed to the conception and design of the study. Romina Bagheri and Ahmad Zolghadriha did the literature search. Afagh Anjomshoaa performed the statistical analysis. Ahmad Zolghadriha and Romina Bagheri wrote the first draft of the manuscript. All authors contributed to manuscript revision, read, and approved the submitted version.

## Disclosure

The lead author Romina Bagheri affirms that this manuscript is an honest, accurate, and transparent account of the study being reported; that no important aspects of the study have been omitted; and that any discrepancies from the study as planned (and, if relevant, registered) have been explained.

## Ethics Statement

This study adhered to ethical guidelines. The protocol was approved by the Ethics Committee of Zanjan University of Medical Sciences (IR.ZUMS.REC.1401.236). Informed consent was obtained from all participants, ensuring the privacy and confidentiality of their information. All procedures followed the ethical standards set forth by the Regional Research Committee and the Declaration of Helsinki (1964 and later amendments).

## Consent

The authors have nothing to report.

## Conflicts of Interest

The authors declare no conflicts of interest.

## Supporting information

Supporting Table S1

## Data Availability

The data that support the findings of this study are available from the corresponding author upon reasonable request.
