# Supplementary material for: Efficacy of Dextromethorphan Augmentation in SSRI‐Resistant Obsessive–Compulsive Disorder: A Randomized Controlled Trial
Source: Health Sci Rep. 2026 May 5;9(5):e72055. doi: 10.1002/hsr2.72055 (PMC13144750; doi:10.1002/hsr2.72055)
Supplement: Supplementary file 1 — Supporting Table S1 [file HSR2-9-e72055-s001.docx]

**Supplementary Table S1.**

*Concomitant SSRI medications and doses at baseline by treatment group*

| **SSRI** | **DXM Group (n=20)** | **Placebo Group (n=20)** |
| --- | --- | --- |
| Fluoxetine (40-80 mg/day) | 7 | 10 |
| Sertraline (150-200 mg/day) | 10 | 8 |
| Paroxetine (40-60 mg/day) | 0 | 0 |
| Fluvoxamine (200-300 mg/day) | 3 | 2 |
| Escitalopram (20-30 mg/day) | 0 | 0 |
| Total | n = 20 | n = 20 |

*Note*: All participants were receiving stable-dose SSRI monotherapy for at least 12 weeks prior to randomization. Doses are reported as daily totals.
